# Molecular Characterization of a Novel Species of *Capillovirus* from Japanese Apricot (*Prunus mume*)

**DOI:** 10.3390/v10040144

**Published:** 2018-03-23

**Authors:** Armelle Marais, Chantal Faure, Sébastien Theil, Thierry Candresse

**Affiliations:** UMR 1332 Biologie du Fruit et Pathologie, INRA, University Bordeaux, CS 20032, 33882 Villenave d’Ornon, France; chantal.faure@inra.fr (C.F.); sebastien.theil@inra.fr (S.T.); thierry.candresse@inra.fr (T.C.)

**Keywords:** Next Generation Sequencing, fruit tree viruses, *Betaflexiviridae*, Mume virus A, *Capillovirus*

## Abstract

With the increased use of high-throughput sequencing methods, new viruses infecting *Prunus* spp. are being discovered and characterized, especially in the family *Betaflexiviridae.* Double-stranded RNAs from symptomatic leaves of a Japanese apricot (*Prunus* *mume*) tree from Japan were purified and analyzed by Illumina sequencing. Blast comparisons of reconstructed contigs showed that the *P. mume* sample was infected by a putative novel virus with homologies to *Cherry virus A* (CVA) and to the newly described *Currant virus A* (CuVA), both members of genus *Capillovirus*. Completion of the genome showed the new agent to have a genomic organization typical of capilloviruses, with two overlapping open reading frames encoding a large replication-associated protein fused to the coat protein (CP), and a putative movement protein (MP). This virus shares only, respectively, 63.2% and 62.7% CP amino acid identity with the most closely related viruses, CVA and CuVA. Considering the species demarcation criteria in the family and phylogenetic analyses, this virus should be considered as representing a new viral species in the genus *Capillovirus*, for which the name of Mume virus A is proposed.

## 1. Introduction

Since the advent of high-throughput sequencing methods in the plant virology field, a number of new viruses infecting *Prunus* spp. have been described [1,2]. In particular, a significant number of new viruses belonging to the *Betaflexiviridae* family have been discovered and characterized, leading to the introduction of two novel genera, *Prunevirus* [3] and *Robigovirus* [4]. More recently, a novel viral species belonging to the family *Secoviridae* has been described in peach, for which the name of Peach leaf pitting-associated virus was proposed [5]. Two marafiviruses in the *Tymoviridae* family were also recently reported from nectarine and peach: the nectarine virus M and the peach virus D, respectively [6,7].

*Prunus mume,* also known as Japanese apricot, belongs to the genus *Prunus* in the family *Rosaceae* and originates from China. It has been cultivated throughout East Asia for fruit production, as well as for ornamental purposes. Besides its cultural significance, *P. mume* is also cultivated for culinary or medicinal uses. To this date, 15 viroids or viral species from families *Pospiviroidae*, *Closteroviridae*, *Bromoviridae*, *Potyviridae*, *Luteoviridae*, *Geminiviridae*, and *Betaflexiviridae* have been described from symptomatic or from symptomless *P. mume* [2,8,9,10,11]. However, due to mixed infections, it is often difficult to associate the presence of a particular virus with the observed symptomatology. As for *Prunus* spp. in general, many of the viral species infecting *P. mume* belong to the *Betaflexiviridae* family. Four genera are thus represented in *P. mume* among the 11 comprised in the family, with eight viruses: *Apricot latent virus* (ApLV) and *Asian prunus virus 1* and *2* (APV1, APV2) (Genus *Foveavirus*), *Peach mosaic virus* (PcMV) and *Apple chlorotic leaf spot virus* (ACLSV) (Genus *Trichovirus*), *Apricot vein clearing associated virus* (AVCaV) (Genus *Prunevirus*), and *Apple stem grooving virus* (ASGV) and *Cherry virus A* (CVA) (Genus *Capillovirus*) [2,12,13].

In the present study, the complete genome sequence of a novel viral species from a Japanese *P. mume* tree showing slight symptoms of diffuse chlorotic spots was determined by a high-throughput sequencing approach. Phylogenetic analyses allow one to conclude that this virus, for which the name of Mume virus A is proposed, belongs to the genus *Capillovirus* in the family *Betaflexiviridae*, extending our knowledge of *Prunus*-infecting viruses.

## 2. Materials and Methods

### 2.1. Plant Materials, Viral Source, and Maintenance

Symptomatic leaves from a Japanese apricot tree (*P. mume*, “PM14”) showing diffuse chlorotic spots on leaves were collected in Japan (Kyoto province) and submitted to high-throughput sequencing analysis. The PM14 isolate was propagated by chip budding on GF305 peach seedlings under level 3 containment greenhouse conditions. Various *Prunus* sources from China, Japan, Czech Republic, Azerbaijan, Kazakhstan, Italy, and France, (*P. mume*, *Prunus cerasus*, *Prunus avium*, *Prunus armeniaca*, *Prunus persica*, *Prunus sibirica*, *Prunus serrulata*, *Prunus salicina*, *Prunus domestica*, *Prunus dulcis*, and *Prunus holosericea*) were screened by Reverse Transcription-Polymerase Chain Reaction (RT-PCR) for the presence of Mume virus A.

### 2.2. Determination of the Herbaceous Host Range of PM14 Isolate

Young leaves of GF305 infected with the PM14 source were used as the inoculum. Leaves were ground (1/4, weight (*wt*)/*v*) in 50 mM potassium phosphate buffer, pH 7.6. Before transmission, activated charcoal (90 mg/mL), nicotine hemisulfate (2% *v*/*v*), and polyvinylpyrrolidone 40 (PVP40, 2% *v*/*v*) were added to the homogenate. Carborundum (400 mesh) was used as the abrasive. The following plants were evaluated as potential hosts: *Chenopodium amaranticolor*, *C. quinoa*, *Nicotiana benthamiana*, *Nicotiana tabacum* cv. Xanthi, *Nicotiana glutinosa*, and *Phaseolus vulgaris* cv. Vedette. The appearance of symptoms was monitored over a three-week period.

### 2.3. Analysis of Double-Stranded RNAs by High-Throughput Sequencing and Completion of the PM14 Isolate Genome Sequence

Double-stranded RNAs (dsRNAs) were extracted from symptomatic leaves of PM14 according to the protocol of Gentit et al. [14] with the modifications introduced by Candresse et al. [15]. The purified dsRNAs were then reverse-transcribed, randomly amplified, and submitted to Illumina sequencing in a multiplexing scheme as described by Candresse et al. [15]. After demultiplexing and quality trimming steps, the reads were assembled using CLC Genomics Workbench 6.5 (http://www.clcbio.com) or a pipeline integrating the Newbler assembler. Contigs were then annotated by BlastN and BlastX comparison with the Genbank database (cut-off *e*-value = 10^−3^). Mapping on reference genomes allowed the ordering of the contigs and the reconstruction of a unique scaffold spanning the viral genome.

To complete the viral genome sequence, primers were designed from the contig sequences and used to amplify PCR products spanning the gaps between contigs (Table 1). The two genome ends were determined by a Rapid Amplification of Complementary DNA (cDNA)Ends strategy for the 5’ end (SMARTER RACE 5′ kit, Takara Bio Europe/Clontech, Saint-Germain-en-Laye, France) and a RT-PCR using the LD-prime and an internal primer for the 3′ end (Table 1) following the protocol described by Youssef et al. [16]. The amplified fragments were submitted to Sanger sequencing (GATC Biotech, Mulhouse, France).

The sequence reported in the present manuscript has been deposited in the GenBank database under accession number MG783575.

### 2.4. Total Nucleic Acid Extraction and RT-PCR Detection of Mume Virus A

Extraction of total nucleic acids (TNAs) was performed according to the protocol 2 described by Foissac et al. [17]. Five microliters of TNAs were submitted to a reverse transcription using a mixture of 2 µM dN6 and 1 µM dT18 as reverse primers and the RevertAid Reverse Transcriptase following the recommendations of the manufacturer (Thermo Fisher Scientific, Illkirch, France). The cDNA was then amplified using 1 µM of Capillo-mume-F1 and Capillo-mume-R1 primers (Table 1) and DyNAzyme II DNA polymerase according to the provider’s instructions (Finnzymes/Thermo Fisher Scientific). Forty cycles were applied, each of 95 °C for 30 s, 61 °C for 30 s, and 72 °C for 30 s, followed by a final extension step of 10 min at 72 °C.

### 2.5. Phylogenetic Analyses

Multiple alignments of nucleotide (nt) or amino acid (aa) sequences were performed using the ClustalW program [18] implemented in MEGA version 6.0 [19]. Genetic distances (p-distances using strict nt or aa identity) were calculated using MEGA 6.0. Phylogenetic reconstructions were performed using the neighbor-joining technique and randomized bootstrapping (1000 replicates) to evaluate the statistical significance of branches.

## 3. Results and Discussion

### 3.1. Illumina Sequencing of Double-Stranded RNAs Purified from the PM14 Japanese Apricot Source

Double-stranded RNAs purified from the PM14 source were sequenced on an Illumina Miseq platform using paired-end sequencing (2 × 250 nt). After demultiplexing, quality trimming, and de novo assembly of the 33,113 reads obtained for that sample, Blast analysis allowed one to identify ten contigs sharing significant identity with known plant viruses. Four contigs integrating 1.6% of total reads were tentatively identified as belonging to a luteovirus (under characterization). The six remaining viral contigs integrated 1.59% of the reads and showed between 71% and 78% nt identity (54% to 83% aa identity for the encoded proteins) with the corresponding regions of two members of the genus *Capillovirus*, CVA or *Currant virus A* (CuVA). These contigs, tentatively identified as belonging to a *capillovirus*, were manually assembled by mapping to CVA genome, in order to create a scaffold of 6542 nt missing the 5′ and 3′ genome ends and containing five internal gaps. The genome sequence of the PM14 isolate was completed by targeted PCR as described in the Materials and Methods section. The assembled complete genome sequence was deposited in the GenBank database under accession number MG783575.

### 3.2. Genome Organization and Phylogenetic Relationships of PM14 Isolate

**Figure 1:**
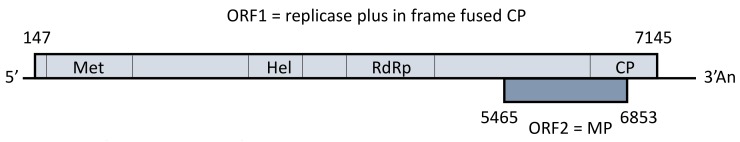
Schematic representation of the genome organization of PM14 isolate. The two predicted open reading frames (ORFs) are represented by boxes, with nucleotide coordinates indicated. Conserved motifs for viral methyltransferase (pfam 01660, Met), viral helicase_1 (pfam 01443, Hel), and RNA-dependent RNA polymerase_2 (pfam 00978, RdRp) domains are shown within ORF1, as well as the coat protein (CP) domain. MP, movement protein. 5′ and 3′ non coding regions are shown by black lanes at both extremities. An: polyA tail.

**Figure 2:**
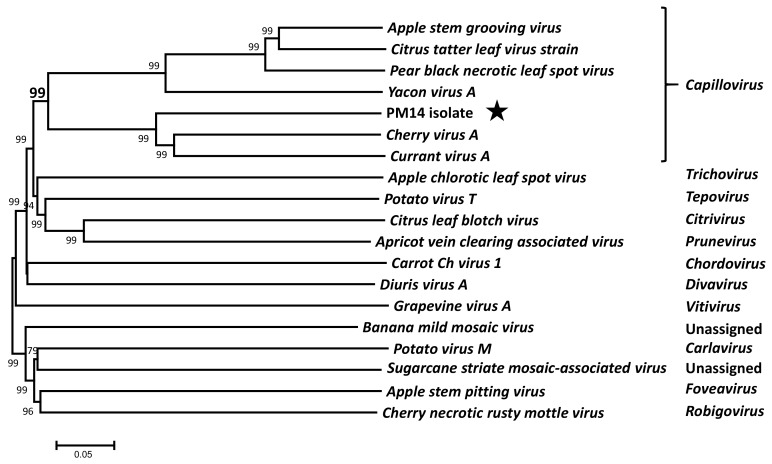
Unrooted phylogenetic tree reconstructed using the complete genome sequences of representative *Betaflexiviridae* members. The tree was constructed using the neighbor-joining method, and the statistical significance of branches was evaluated by bootstrap analysis (1000 replicates). Bootstrap values above 70% are shown. The scale bar represents 5% divergence between sequences. Sequences retrieved from GenBank are NC001749 *Apple stem grooving virus*; AY646511 *Citrus tatter leaf virus*; AY596172 *Pear black necrotic leaf spot virus*; NC030657 *Yacon virus A*; NC003689 *Cherry virus A*; NC029301 *Currant virus A*; NC001409 *Apple chlorotic leaf spot virus*; NC011062 *Potato virus T*; NC003877 *Citrus leaf blotch virus*; NC023295 *Apricot vein clearing associated virus*; NC025469 *Carrot Ch virus 1*; NC019029 *Diuris virus A*; NC003604 *Grapevine virus A*; NC002729 *Banana mild mosaic virus*; NC001361 *Potato virus M*; NC003870 *Sugarcane striate mosaic-associated virus*; and NC002468 *Cherry necrotic rusty mottle virus*. The genus to which each virus belongs is indicated at the right. The PM14 isolate is indicated by a black star.

The genome of the PM14 isolate has a size of 7644 nt, excluding the polyA tail. It encodes two overlapping open reading frames (ORFs), with an overall genome organization similar to that of *capilloviruses* (Figure 1). A phylogenetic tree reconstructed using the complete genome sequences of representative members of the *Betaflexiviridae* family shows that the new virus clusters with other *capilloviruses* with a highly significant bootstrap value (99% in Figure 2) and, more specifically, that it forms a sub-cluster together with CVA and CuVA.

The ORF1 (2333 aa) encodes a large replication-associated protein (aa 1–2105) fused to the coat protein (CP, aa 2106–2333). Three conserved domains typical for *Betaflexiviridae* replicases are found within the ORF1 protein: a viral methyltransferase domain (pfam 01660, aa 43-351), a viral helicase 1 domain (pfam 01443, aa 812–1089), and a RNA-dependent RNA polymerase 2 domain (pfam 00978, aa 1213-1541). No AlkB domain (2OG-FeII-Oxy-2-domain) has been identified within the ORF1 protein, as for most of *Capillovirus* members, with the exception of CuVA ORF1 in which AlkB domain was found between aa 623 and 736 [20,21]. The second ORF encodes a putative movement protein (MP, 463 aa). The two non-coding regions (NCR) located at the 5’ and 3’ ends of the genome are 146 and 499 nt long, respectively. Both NCRs are thus significantly longer than those of other capilloviruses, which are reported to be between 35 and 93 nt (5’ NCR of ASGV and CuVA, respectively) and between 122 and 303 nt (3’ NCR of Yacon virus A and CVA, respectively). Interestingly, the size of the 5’ and 3’ NCRs seems to be correlated with the clustering of CVA, CuVA, and the PM14 isolate in the same phylogenetic group, and the same applies to the size of the various proteins (Table 2).

**Figure 3:**
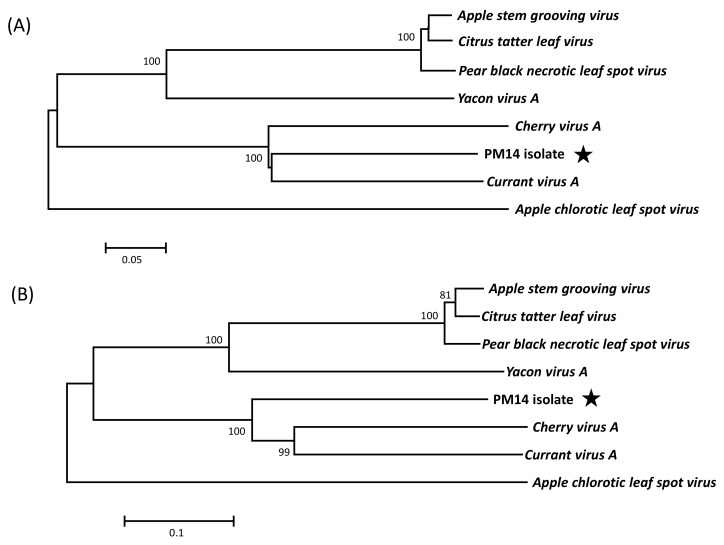
Neighbor joining phylogenetic trees reconstructed using the amino acid sequences of the coat protein (**A**) or movement protein (**B**) from *Capillovirus* members. *Apple chlorotic leaf spot virus* (NC001409 *Trichovirus* genus) was included as outgroup. The trees were constructed in Mega 6.0 using a strict amino acid identity distance. Bootstrap values above 70% (1000 replicates) are shown. The scale bars represent 5% (**A**) or 10% (**B**) divergence. Sequences retrieved from GenBank are the same as in Figure 2. The PM14 isolate is indicated by a black star.

Phylogenetic analyses based on alignments of CP or MP sequences from *Capillovirus* members confirm a clear clustering of the PM14 isolate with CVA and CuVA, with highly significant bootstrap values (100%) (Figure 3A,B). The same clustering is also obtained using replicase sequences (data not shown). To complete this analysis, sequence identities were calculated among *Capillovirus* members for the replicase, the CP and the MP (Table 2). The level of identity between these proteins is distant, with, at best, only 65.4% of aa identity between PM14 isolate and CuVA in the CP, and 54.4% with CVA in the replicase. Considering the accepted species demarcation molecular criteria for the family *Betaflexiviridae*, which are of 72% nt identity (or 80% aa identity) in the replicase and CP genes [21], the PM14 isolate should be considered as a novel species in the genus *Capillovirus*. We therefore propose the name of Mume virus A (MuVA) for this species.

### 3.3. Biological Characterization and Incidence of Mume virus A

Despite significant efforts to transmit MuVA to various herbaceous plants, none of the plant species tested could be successfully inoculated. No local or systemic symptoms appeared on inoculated plants, and RT-PCR assays performed to detect MuVA were all negative. MuVA was, however, successfully graft-transmitted to peach seedlings (GF305 indicator). However, no symptoms could be identified on leaves or in growth habit of the infected peach plants (data not shown). The initial tree in which MuVA was detected showed diffuse chlorotic spots on leaves. However, it is co-infected by a potentially novel luteovirus (currently under characterization), so that it is difficult to assess the role of MuVA in the symptomatology observed. Moreover, due to the dsRNA sequencing strategy used in the present study, the potential contribution in the observed symptoms of another agent such as a DNA virus or a viroid cannot be excluded. Among the capilloviruses described so far, four are known to infect fruit trees, which represent in fact only two viral distinct species, CVA and ASGV, since *Citrus tatter leaf virus* (CTLV) and *Pear black necrotic leaf spot virus* (PBNLSV) are recognized as citrus and pear isolates of ASGV, respectively [22,23]. CVA infection is very generally latent in its cherry hosts, and no symptoms are associated with ASGV infection on apple cultivars. Both viruses have been shown to be transmitted by grafting, but only ASGV was successfully transmitted to various herbaceous host species [24,25]. No natural vector is known so far for capilloviruses, and CVA and ASGV are not known to be epidemic and are apparently transmitted in the field only by grafting and by planting infected material [12,24]. To assess the incidence of MuVA, a total of 285 samples from various *Prunus* species originated from seven different countries were screened by a specific RT-PCR test (Capillo-mume-F1/R1; Table 1). No MuVA-infected sample was detected, even among *P. mume* samples collected in East Asian countries (China and Japan), suggesting a limited prevalence of MuVA. Further investigations are necessary to evaluate the distribution, prevalence, and potential pathogenicity of this new member of the *Capillovirus* genus.

## Figures and Tables

**Table 1:** Primers used in this study to complete the genome sequence of the PM14 isolate.

Primer Name	Sequence (5′-3′)	Genome Coordinates	Amplicon Size (nt)
Capillo-mume-5Race2 ^#^	CCTTGCATGGTTGTTGTTGAAGTCCTCCC	251–223	252
Capillo-mume-F1	AACAACAACCATGCAAGGTTTGAG	234–257	503
Capillo-mume-R1	GCTAGAACACACTTAGGCCGCAA	736–714	
Capillo-mume-F2	GGAATGTTGATACATACAGACA	1629–1650	742
Capillo-mume-R2	CGTCTGAGCCTAATCCATACAC	2370–2349	
Capillo-mume-F3	TGGATTTATTGAACTTCTCATAC	2831–2853	406
Capillo-mume-R3	CGTCACAATCACACCAAATCTG	3236–3215	
Capillo-mume-F6	GATGTACGAGGATTCAGTGG	4034–4053	451
Capillo-mume-R6	AATGAGGGAGTTAGAAACACC	4484–4464	
Capillo-mume-R7 ^~^	ACCAACTGTTATGACAGATTC	5117–5097	1084
LD-PolyT ^@^	CACTGGCGGCCGCTCGAGCATGTAC(T)25NN		
Capillo-mume-LD	GAGCACCATTGGAGGGTGTGT	7462–7482	183
LD-Prime	CACTGGCGGCCGCTCGAGCATGTAC		

^#^ This reverse primer was used in conjunction with the universal primer provided by the 5’ RACE kit (Takara Bio Europe/Clontech, Saint-Germain-en-Laye, France); ^~^ This reverse primer was used in conjunction with the Capillo-mume-F6 primer; ^@^ This primer was used for the complementary DNA synthesis prior amplification with the primer pair Capillo-mume-LD and LD-Prime.

**Table 2:** Percentages of identity between the proteins encoded by the genome of the PM14 isolate and the corresponding proteins of *Capillovirus* members ^#^.

Virus ^@^	Percent Amino Acid Identity (Size [aa])		
	Replicase (2105)	Coat Protein (228)	Movement Protein (463)
CuVA	53.7% (2296)	**65.4**% (229)	53.3% (462)
CVA	**54.4**% (2112)	62.7% (232)	**53.7**% (463)
ASGV	25.7% (1868)	32.1% (238)	29.1% (321)
YVA	24.6% (1868)	30.2% (215)	26.2% (324)

^#^ Sizes in amino acids (aa) of the proteins are indicated in parentheses. For each protein, the highest identity level is highlighted in bold. ^@^ Acronyms used: CuVA = *Currant virus A*, CVA = *Cherry virus A*, ASGV = *Apple stem grooving virus*, YVA = *Yacon virus A*.
